# Sensory Transduction Channel Subunits, *tax-4* and *tax-2*, Modify Presynaptic Molecular Architecture in *C. elegans*

**DOI:** 10.1371/journal.pone.0024562

**Published:** 2011-09-07

**Authors:** Andrew B. Hellman, Kang Shen

**Affiliations:** 1 Department of Biology, Stanford University, Stanford, California, United States of America; 2 Howard Hughes Medical Institute, Stanford University, Stanford, California, United States of America; 3 Department of Pathology, Stanford University, Stanford, California, United States of America; Max-Planck-Institut für Neurobiologie, Germany

## Abstract

During development, neural activity is important for forming proper connections in neural networks. The effect of activity on the gross morphology and synaptic strength of neurons has been well documented, but little is known about how activity affects different molecular components during development. Here, we examine the localization of four fluorescently-tagged presynaptic proteins, RAB-3, SNG-1/synaptogyrin, SYD-2/Liprin-α, and SAD-1/SAD kinase, in the *C. elegans* thermosensory neuron AFD. We show that *tax-4* and *tax-2*, two genes that encode the cyclic nucleotide-gated channel necessary for sensory transduction in AFD, disrupt the localization of all four proteins. In wild-type animals, the synaptic vesicle (SV) markers RAB-3 and SNG-1 and the active zone markers SYD-2 and SAD-1 localize in a stereotyped, punctate pattern in the AFD axon. In *tax-4* and *tax-2* mutants, SV and SYD-2 puncta are more numerous and less intense. Interestingly, SAD-1 puncta are also less intense but do not increase in number. The change in puncta number can be rescued cell-autonomously in AFD. These results suggest that sensory transduction genes *tax-4* and *tax-2* are necessary for the proper assembly of presynapses.

## Introduction

Neural activity sculpts the nervous system at multiple stages of development and is critical for memory formation [1], [2]. Most studies have used the stabilization of axons and dendrites or a change in synaptic strength to measure activity-dependent cellular responses. Few have observed how the molecular architecture of presynaptic specializations, independent of axon and dendrite morphology, is affected by activity. The localization of the presynaptic protein syanpsin has been shown to be acutely activity-dependent [3]–[5] but the localization of other presynaptic proteins in sensory activity-dependent development has not been studied. Understanding how individual molecular components respond to activity during development provides a more detailed picture of what occurs during activity-dependent processes.

In the vertebrate visual system, both spontaneous activity and sensory input contribute to the refinement of neural inputs [6], [7]. Light sensation in vertebrates, and odor sensation, depend on a signaling cascade that includes hetero-oligomeric cyclic nucleotide-gated (CNG) channels and cyclases, which generate cyclic nucleotides [8], [9]. The nematode *C. elegans* is able to sense environmental temperature [10], a process that requires the sensory neuron AFD, a CNG channel and its upstream cyclases. Ablation of AFD causes defects in thermotaxis behavior in *C. elegans* [11]. Mutations in the *tax-4* or *tax-2* gene, which encode the alpha- and beta- subunits, respectively, of a cGMP-gated channel, cause thermotaxis defects [12]–[14]. The genes *gcy-8*, *gcy-18*, and *gcy-23* encode three redundant guanylate cyclases that are expressed specifically in AFD and are necessary for thermotaxis behavior [15]. Calcium imaging and electrophysiology experiments have demonstrated that *tax-4* mutants lack a response to temperature in AFD [16], [17]. Thus, *tax-4* mutations disrupt thermosensory input into AFD.

In this paper, we observe that the pattern of synapses in the *C. elegans* thermosensory neuron AFD is strikingly stereotyped. Mutations in the AFD sensory machinery cause mislocalization of multiple presynaptic proteins. The number of presynaptic puncta increases for synaptic vesicle (SV) markers and the active zone (AZ) marker SYD-2 but decreases for the active zone marker SAD-1. The changes in puncta number can be rescued cell-autonomously. These observations demonstrate that the *tax-4* and *tax-2* genes, which are essential for sensory activity, are necessary for the proper development of presynaptic specializations.

## Results

### Multiple presynaptic proteins localize in a stereotyped pattern in AFD

**Figure 1:**
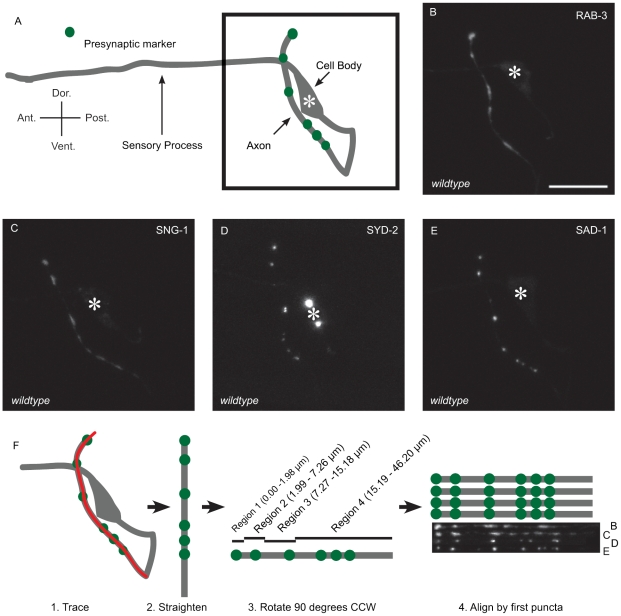
Multiple presynaptic components in AFD localize in a stereotyped pattern. (A) Schematic of AFD. A sensory process, referred to as the dendrite, extends anteriorly from the cell body. A second process, referred to as the axon, extends posteriorly and ventrally, then fasciculates with the nerve ring, where it forms synapses onto its sole post-synaptic partner, the interneuron AIY. Green dots, presynaptic specializations. RAB-3 (B), SNG-1 (C), SYD-2 (D), and SAD-1 (E) localization in AFD. Asterisk, cell body; scale bar, 10 µm. (F) Image processing workflow. Axons of confocal projections were traced (red line) and straightened using ImageJ. Straightened traces were then rotated 90 degrees counter clockwise and aligned by the center of the dorsal-most puntum of each animal. Regions were assigned based on stereotyped localization of presynaptic markers.

*C. elegans* AFD consists of a pair of bilaterally symmetrical sensory neurons, AFDL and AFDR. The cell bodies, located in the lateral ganglion, each extend two processes. One projects anteriorly into the tip of the nose, where the molecular machinery involved in sensing temperature, including TAX-4 and TAX-2, is localized [12], [13], [15]. The second process projects into the nerve ring, where eight to thirteen synapses are formed from AFD onto the interneuron AIY (Fig. 1A) and AFD receives synaptic inputs from interneurons and other sensory neurons [18].

To visualize the presynaptic specializations of AFD, we drove expression of fluorescently-tagged presynaptic proteins with the AFD-specific promoter of the *gcy-8* gene [19]. The SV markers RAB-3 and SNG-1 localized in discrete puncta along the axon of AFD (Fig. 1B–C). The AZ markers SYD-2 and SAD-1 also localized as discrete puncta along the axon of AFD (Fig. 1D–E). In each of the markers, the number of puncta in the axon increases throughout development (Fig. S1), suggesting synapses are added or proteins are reallocated during growth.

These markers revealed a consistent pattern of presynaptic specializations in AFD. To visualize this pattern on a population level, we took confocal stacks of at least 20 L4 animals. From the stacks, we created a maximum intensity projection, straightened the axons using ImageJ software, and then aligned the traces by the dorsal-most punctum of each animal (Fig. 1F). Using this method, we found that there was a distinct and stereotyped pattern of presynaptic specializations in AFD. We also found that all SNG-1 puncta co-localize with RAB-3 and most SYD-2 puncta co-localize with RAB-3 (Fig. S2 and S3). The existence of a synaptic pattern is surprising considering the fact that all the presynaptic terminals from AFD have interneurons AIY as their synaptic partners.

To quantitatively represent this distribution pattern, we divided the AFD axons into four major landmarks or regions. The first region (R1) is a dorsal-most punctum, which is determined during imaging (see Experimental Procedures). The second region (R2) is 1.99–7.26 µm from the center of the dorsal-most punctum, and usually consists of one punctum. The third region (R3) is 7.27–15.18 µm from the dorsal-most punctum. The fourth region (R4) consists of a “ventral run” of puncta and is defined as 15.19–46.20 µm from the initial dorsal-most punctum. This fourth region contains the least stereotyped pattern of puncta. All markers displayed patterns consistent with this classification, and the observed pattern correlates well with the reconstruction of the nervous system by electron microscopy [18]. These data suggest that presynaptic markers RAB-3, SNG-1, SYD-2, and SAD-1 label presynapses in AFD.

### Synaptic vesicle markers mislocalize in tax-4 and tax-2 mutants

#### RAB-3

In order to identify molecular mechanisms for synapse formation, we performed a semi-clonal F2 screen to identify animals displaying an altered distribution of presynaptic specializations. We isolated *wy349* and used single nucleotide polymorphism (SNP) mapping [20], [21] to map the mutation to the middle of chromosome III, where *tax-4* is located. *wy349* failed to complement the canonical *tax-4* allele *p678*, and DNA sequencing revealed *wy349* to be an Asn to Lys conversion in the fourth transmembrane domain of *tax-4*. *wy349* phenocopied *tax-4(p678)*, which causes a Gln to Amber Stop mutation before the first transmembrane domain. *p678* was used for the remainder of the study because it is well-characterized and behaves as a putative null [13].

**Figure 2:**
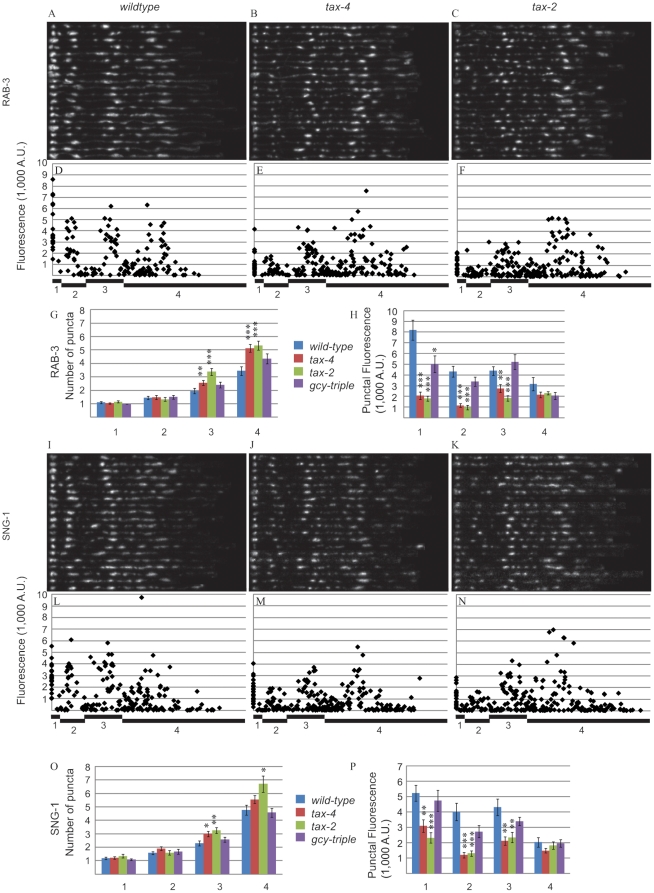
*tax-4* and *tax-2* mutants have altered distributions of the synaptic vesicle proteins, RAB-3 and SNG-1. (A–C) Traces of the RAB-3 marker in 20 wild-type (A), *tax-4* (B), and *tax-2* (C) animals are shown. Each row represents one animal. (D–F) Fluorescence values for each RAB-3 punctum are plotted versus the distance from the center of the dorsal-most punctum in wild-type (D), *tax-4* (E), and *tax-2* (F) animals. Regions are noted below the x-axis. (G) Quantification of the average number of RAB-3 puncta per animal in each region. (H) Quantification of the average intensity of RAB-3 puncta per animal in each region. (I–K) Traces of the SNG-1 marker in 20 wild-type (I), *tax-4* (J), and *tax-2* (K) animals are shown. Each row represents one animal. (L–N) Fluorescence values for each SNG-1 punctum are plotted versus the distance from the center of the dorsal-most punctum in wild-type (L), *tax-4* (M), and *tax-2* (N) animals. (O) Quantification of the average number of SNG-1 puncta per animal in each region. (P) Quantification of the average intensity of SNG-1 puncta per animal in each region. ***, p<0.001; **, p<0.01; *, p<0.05 by student t-test.

In wild-type animals, GFP::RAB-3 displayed the stereotypical AFD presynaptic pattern (Fig. 2A). When we plotted the fluorescence intensity of puncta against their positions along the axon, four peaks were prominent in R1, R2, R3, and R4 (Fig. 2D). In *tax-4* and *tax-2* mutants, RAB-3 exhibited an altered distribution. While the four peaks were still identifiable, the sizes of the peaks were different compared to wild-type (Fig. 2B, C). R1, R2, and R3 peaks were lower in the mutants. Additionally, there was an increase in the number of dim puncta in R2, R3, and R4 (Fig. 2E, F).

We quantified the average number and intensity of puncta in each region and found significant differences between wild-type and the mutants *tax-4* and *tax-2*. In R1 and R2, no significant difference was observed in the number of puncta between wild-type and *tax-4*, *tax-2*, or *gcy-23 gcy-8 gcy-18* triple mutants (*gcy-triple*), which lack three guanylyl cyclases that redundantly act upstream of *tax-4* and *tax-2* [15]. In R3 and R4, *tax-4* and *tax-2* had more puncta than wild-type animals, but *gcy-triple* animals did not differ (Fig. 2G). Compared to wild-type, the average intensity of puncta decreased dramatically in R1, R2, and R3 in *tax-4* and *tax-2* animals. However, no significant difference was observed between wild-type and *tax-4* and *tax-2* in R4. *gcy-triple* animals' puncta were dimmer only in R1 (Fig. 2H). These results suggest that without *tax-4* or *tax-2*, either the number of small presynaptic specializations increases or RAB-3 molecules form aggregates in the axon of AFD.

#### SNG-1

To confirm that SVs and not only RAB-3 biology is affected, we studied the effect of sensory mutants on another SV protein, SNG-1, and found that it acted similarly to RAB-3. Wild-type, *tax-4*, *tax-2*, and *gcy-triple* animals displayed presynaptic patterns similar to RAB-3 (Fig. 2I-K). R1, R2, and R3 peaks in the SNG-1 marker were lower in *tax-4* and *tax-2* than in wild-type (Fig. 2L–M). In R1 and R2, there was no significant difference in the number of puncta between wild-type, *tax-4*, and *tax-2*. In R3, *tax-4* and *tax-2* had more puncta than wild-type, but in R4 only *tax-2* had significantly more puncta than wild-type, though *tax-4* trends upward (Fig. 2O). *gcy-triple* mutants were not significantly different from wild-type in any regions.

The trend of average puncta intensity in the SNG-1 marker was also similar to that of RAB-3. In R1, R2, and R3, average puncta intensity decreased in *tax-4* and *tax-2* animals compared to wild-type. In R4, no significant difference was observed between *tax-4*, *tax-2*, and wild-type. *gcy-triple* mutants were not significantly different from wild-type in any regions. The similarities between the RAB-3 and SNG-1 markers in *tax-4* and *tax-2* compared to wild-type suggest that SV localization, not the localization of individual proteins, is affected by mutations in the cyclic nucleotide-gated channel subunits. Indeed, RAB-3 and SNG-1 co-localize in mutant animals (Fig. S2).

### Active zone markers mislocalize in tax-4 and tax-2 mutants

#### SYD-2

**Figure 3:**
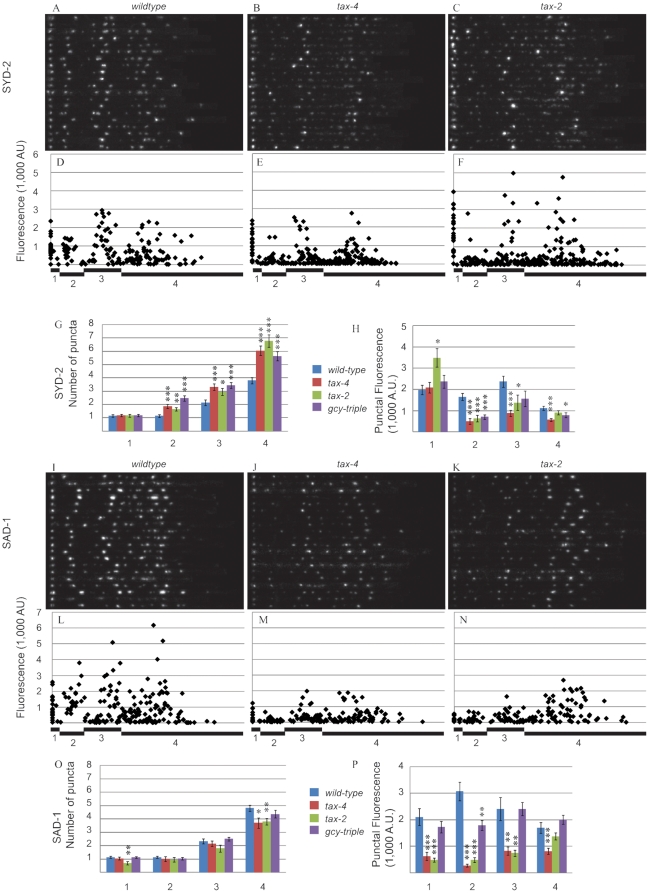
*tax-4* and *tax-2* mutants have altered distributions of the active zone proteins, SYD-2 and SAD-1. (A–C) Traces of the SYD-2 marker in 20 wild-type (A), *tax-4* (B), and *tax-2* (C) animals are shown. Each row represents one animal. (D–F) Fluorescence values for each SYD-2 punctum are plotted versus the distance from the center of the dorsal-most punctum in wild-type (D), *tax-4* (E), and *tax-2* (F) animals. Regions are noted below the x-axis. (G) Quantification of the average number of SYD-2 puncta per animal in each region. (H) Quantification of the average intensity of SYD-2 puncta per animal in each region. (I–K) Traces of the SAD-1 marker in 20 wild-type (I), *tax-4* (J), and *tax-2* (K) animals are shown. Each row represents one animal. (L–N) Fluorescence values for each SAD-1 punctum are plotted versus the distance from the center of the dorsal-most punctum in wild-type (L), *tax-4* (M), and *tax-2* (N) animals. (O) Quantification of the average number of SAD-1 puncta per animal in each region. (P) Quantification of the average intensity of SAD-1 puncta per animal in each region. ***, p<0.001; **, p<0.01; *, p<0.05 by student t-test.

We next asked whether only SVs were being affected or other presynaptic proteins were also mis-localized. To answer this, we analyzed the localization of the AZ protein SYD-2 in wild-type, *tax-4*, and *tax-2*. In the SYD-2 marker, wild-type animals displayed a stereotypical AFD synaptic pattern with four prominent peaks (Fig. 3A and D). Similar to the behavior of SV markers, the SYD-2 puncta in R2 and R3 were dimmer in *tax-4* and *tax-2*. In addition, there was an increase in the number of dim puncta in these regions (Fig. 3B, C and 3E, F).

When we compared the wild-type animals to *tax-4* and *tax-2*, the number of SYD-2 puncta also showed similar changes to those of SV markers. In R2, R3, and R4, the number of SYD-2 puncta in *tax-4* and *tax-2* was greater than wild-type. Interestingly, this was also the case for the *gcy-triple* animals, which is a different result than that observed for the SV markers (Fig. 3G). In R1, no significant difference was observed.

Trends for the average puncta intensity in the SYD-2 marker were similar to those of SV markers in R2 and R3 but different in R1 and R4. In R2 and R3, average puncta intensity decreased in *tax-4* and *tax-2* relative to wild-type, similar to SV markers. In contrast to SV markers, average puncta intensity did not decrease in R1 of *tax-4* and *tax-2* compared to wild-type. In R4, average puncta intensity of *tax-4*, but not *tax-2*, was lower than wild-type. While R4 appears different in the SYD-2 marker than the SV markers, it is worth noting that the average puncta intensity of SV markers trends lower in *tax-4* and *tax-2* mutants than wild-type.

Together with the analysis of the SV markers, these data suggest that more presynaptic specializations are being formed. Another possibility is that SYD-2, which is thought to be an important scaffolding molecule, is forming ectopic aggregates, which then recruit SVs in AFD in *tax-4* and *tax-2* mutants. Indeed, RAB-3 and SYD-2 show a high degree of co-localization in *tax-4* mutants (Fig. S3). The lack of a mutant phenotype for SV markers in *gcy-triple* mutants and the presence of one in the SYD-2 marker suggest that the SYD-2 mis-localization is not sufficient to cause mis-localized SVs.

#### SAD-1

In order to further characterize the extra puncta found in *tax-4* and *tax-2* mutants, we analyzed the localization of another AZ protein. If the mutants affect multiple AZ markers similarly, it would support the hypothesis that the puncta represent presynaptic specializations as opposed to a redistribution of a subset of presynaptic components. Contrary to this hypothesis, we found that the SAD-1 marker was affected differently in *tax-4* and *tax-2* mutants than the SV and SYD-2 markers. Though puncta were dimmer in *tax-4* and *tax-2* mutants relative to wild-type, which is consistent with the other markers, the number of SAD-1 puncta did not increase, which is the opposite phenotype of the other markers (Fig. 3I–N).

Unlike SYD-2, the number of SAD-1 puncta did not increase in any region in *tax-4*, *tax-2*, or *gcy-triple* mutants compared to wild-type; in fact, the number of puncta decreased in R4 in *tax-4* and *tax-2* (Fig. 3O). The average puncta intensity in all regions significantly decreased in *tax-4* and in R1, R2, and R3 in *tax-2* compared to wild-type (Fig. 3P). *Gcy-triple* mutants only differed from wild-type in R2.

This difference between the SYD-2 and SAD-1 markers suggests that the additional SV and SYD-2 puncta that we observed in *tax-4* and *tax-2* mutants may not contain the full complement of presynaptic proteins. Thus, rather than fully developed synapses, the extra puncta may be aggregates of SYD-2 and SVs.

### Tax-4 acts cell autonomously

In addition to AFD, *tax-4* and *tax-2* are expressed in a number of sensory neurons, some of which are connected directly or via interneurons to AFD; thus, the loss of *tax-4* and *tax-2* in other neurons may cause the changes observed AFD. One possible way for *tax-4* and *tax-2* to affect the placement of synaptic material along the axon is to disrupt the fasciculation of AFD and AIY axons. However, at the light microscope level, we noted no fasciculation defects or abnormalities in the morphology of AFD or AIY in *tax-4* mutants (Fig. S4).

We then tested the hypothesis that *tax-4* functions cell autonomously in the AFD neurons to influence its synaptic pattern. We drove expression of the *tax-4* cDNA specifically in AFD using the *gcy-8* promoter and counted the number of puncta in wild-type animals, *tax-4* animals, and *tax-4* animals with the rescue construct. The total number of puncta within the axon serves as a robust measure of the phenotype (Fig. S5). L4 stage animals were used for all three genotypes in RAB-3, SYD-2, and SAD-1 markers. Given the well-documented relationship between *tax-4* and *tax-2* and the strong phenocopy observed in the AFD system presented in Figures 2 and 3, we did not test *tax-2* animals for cell-autonomy.

**Figure 4:**
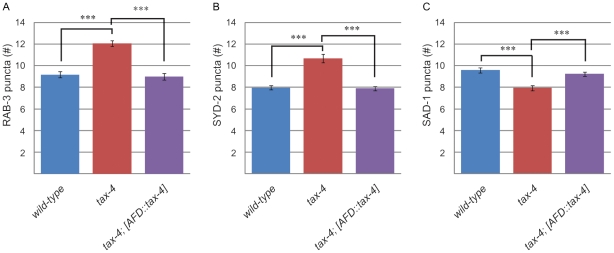
Cell-autonomous rescue of *tax-4*. (A) Number of RAB-3 puncta in 50 or more wild-type animals, *tax-4* animals, and *tax-4* animals expressing *tax-4* specifically in AFD. (B) Number of SYD-2 puncta in 50 or more wild-type animals, *tax-4* animals, and *tax-4* animals expressing *tax-4* specifically in AFD. (C) Number of SAD-1 puncta in 50 or more wild-type animals, *tax-4* animals, and *tax-4* animals expressing *tax-4* specifically in AFD.

For all three markers, the altered number of puncta in the *tax-4* mutants was rescued by the introduction of AFD-specific *tax-4* cDNA. In the RAB-3 marker (Figure 4A), wild-type animals had 9.18±1.92 puncta (n = 50), whereas *tax-4* mutants had 12.06±1.83 puncta (n = 50, p<0.001 compared to wild-type). *tax-4* mutants carrying the AFD-specific *tax-4* rescue construct had 8.98±2.25 puncta (n = 53, p<0.001 compared to *tax-4* and p = 0.63 compared to wild-type). In the SYD-2 marker (Figure 4B), wild-type animals had 7.96±1.37 puncta (n = 50, whereas *tax-4* mutants had 10.68±2.70 puncta (n = 50, p<0.001 compared to wild-type). *tax-4* mutants carrying the AFD-specific *tax-4* rescue construct had 7.90±1.46 puncta (n = 50, p<0.001 compared to *tax-4* and p = 0.83 compared to wild-type). In the SAD-1 marker (Figure 4C), wild-type animals had 9.58±1.68 puncta (n = 50), whereas *tax-4* mutants had 7.94±1.81 puncta (n = 50, p<0.001 compared to wild-type). *tax-4* mutants carrying the AFD-specific *tax-4* rescue construct had 9.23±1.49 puncta (n = 51, p<0.001 compared to tax-4 and p = 0.63 compared to wild-type). Thus, *tax-4* acts cell autonomously in AFD, suggesting that the loss of *tax-4* in non-AFD neurons does not cause the change in synaptic puncta number observed in *tax-4* mutants.

## Discussion

Neural activity is necessary for the proper development of many neural circuits, including the vertebrate visual system [7], the *Drosophila* and mouse neuromuscular junction [22], and the whisker barrel cortex of rats [23]. How experience affects the molecular composition of the presynaptic specializations is not well understood. Here, we report that mutation of *tax-4* and *tax-2*, genes required for sensory activity in a *C. elegans* neuron, changes presynaptic components in a complex manner. It affects different presynaptic terminals of the same neurons differently and affects various presynaptic proteins distinctively.

### Stereotyped localization pattern of AFD presynaptic structures

We show that AFD presynaptic structures form in a stereotyped pattern, which begs the question of why such a pattern exists. One possible reason is that AFD connects to different post-synaptic partners that contact AFD in specific regions, leading to AFD's stereotyped presynaptic pattern. This model must be discarded based on electron micrograph reconstruction studies that reveal AIY is AFD's only post-synaptic partner [18]. Another possible reason for AFD's stereotyped pattern is that “micro-circuits” play an important role in *C. elegans* networks and affect synaptic patterning. The *C. elegans* nervous system lacks voltage-gated sodium channels and therefore axons of *C. elegans* might be isopotential. Additionally, in many neurons, including AIY, dendritic inputs are not spatially separated from axonal outputs by a cell body, as they are in most well-studied vertebrate neurons; individual “axons” sometimes have post-synapses in close proximity to presynapses. This gives rise to the intriguing possibility that the precise location of AFD-AIY synapses may be important because AIY must locally relay synaptic inputs to synaptic outputs.

Consistent with this idea is the fact that AIY outputs are spatially segregated within the axon. According to EM reconstruction of the nervous system [18], AIY synapses onto the neuron HSN only near AFD R3 inputs and onto the AWA, RIA, and RIB neurons only near AFD R4 inputs. Thus, only R3 contains an AFD-AIY-HSN circuit, whereas only R4 contains an AFD-AIY-RIA circuit. These micro-circuits may require precise placement of synapses along an axon. Thus, the specific locations of synapses within the axon may be stereotyped because the AFD presynaptic specializations are coordinated both by AIY and its downstream neurons.

### Complex changes of synaptic vesicle and active zone markers

While all of the presented presynaptic markers have dimmer puncta, there are subtle, intriguing differences between the markers. One difference is between the two AZ markers, SYD-2, a scaffolding protein, and SAD-1, a serine/threonine kinase: in *tax-4* and *tax-2* mutants, the number of SYD-2 puncta increases but the number of SAD-1 puncta decreases. This suggests that many of the puncta observed in the SYD-2 marker lack at least one component of mature synapses. It also suggests that SYD-2 is not sufficient to recruit SAD-1 in *tax-4* and *tax-2* mutants. Though it is not sufficient, it is partially necessary; in AFD, *syd-2* mutants have fewer SAD-1 puncta than wild-type animals, suggesting SYD-2 plays a role in recruiting SAD-1 (data not shown). However, the presence of SAD-1 puncta in the absence of *syd-2* suggests that another, yet unidentified, molecule also recruits SAD-1 to the synapse. In *tax-4* and *tax-2* mutants, this molecule may be sequestering SAD-1. Another alternative explanation for the excessive SYD-2-positive-SAD-1-negative puncta is that they represent non-functional aggregates of presynaptic material. Unfortunately, due to the lack of presynaptic functional assays in vivo, it is currently not possible to directly test this hypothesis.

Another point of interest is that some markers are affected differently depending on region. In R2, R3, and R4, SV, SAD-1, and SYD-2 puncta intensity decreases when comparing *tax-4* and *tax-2* animals to wild-type. However, in R1, SV and SAD-1 puncta intensity decreases but SYD-2 levels remain unchanged, demonstrating that the relative abundance of SYD-2 does not directly determine the abundance of other presynaptic proteins at the synapse. R1 differs from the other regions in that it is located at the terminus of the axon, which may diminish the chances of SVs reaching it. In *tax-4* mutants, the increased number of SYD-2 puncta may represent an increased number of potential docking points for UNC-104/Kif1a-transported SVs. The net result would be that fewer SVs make it to the terminus of AFD. Thus, while SYD-2 levels at the terminus in *tax-4* mutants remain the same as wild-type animals, SV levels decrease.

We also observed that *gcy-triple* does not phenocopy *tax-4* and *tax-2* in all of the markers tested. Ramot et al. [17] show that, similar to *tax-4* and *tax-2* mutants, *gcy-triple* mutants lack an electorphysiological response to increases in temperature. This result suggests that the synaptic protein localization defect we observe is independent of AFD's response to increases in temperature. Additionally, our attempts to silence AFD, which included genetic manipulations of voltage-gated calcium and potassium channels and raising animals at lower or constant temperatures, were unable to induce a *tax-4* phenotype; attempts to rescue the *tax-4* phenotype by overexpressing a voltage-gated sodium channel also failed. It is important to note that these experiments do not conclusively exclude the possibility that sensory activity is causing the phenotype as none of these methods have been shown to silence or stimulate AFD to the extent that *tax-4* and *tax-2* do.

One hypothesis to explain the difference between *gcy-triple* and *tax-4* and *tax-2* phenotypes would be the following: Because the guanylyl cyclases involved in AFD thermosensation are expressed solely in AFD [19] and *tax-4* and *tax-2* are expressed in multiple neurons, other neurons could influence synaptic protein localization of SV and SAD-1 proteins in AFD. However, the *tax-4* defect can be rescued cell autonomously in AFD, ruling out this hypothesis. Thus, one possible explanation that must be considered is that *tax-4* and *tax-2* affect the localization of presynaptic proteins via a mechanism independent of sensing increases in temperature. Such a model would require an upstream pathway independent of *gcy-8*, *gcy-18*, and *gcy-23*. Inada et al. [15] state that a fourth guanylyl cyclase, *gcy-12*, is expressed in AFD but does not appear to play a major role in thermotaxis behavior. Thus, it is possible that *gcy-12* or a yet unidentified cyclase may activate *tax-4* and *tax-2* in response to a stimulus different than that for which *gcy-8*, *gcy-18*, and *gcy-23* have been tested.

## Methods

### Strains and Genetics

Worms were raised on NGM plates seeded with OP50 E. coli at 25°C. PR678 *tax-4(p678)III*, PR671 *tax-2(p671)I*, and IK597 *gcy-23(nj37) gcy-8(oy44) gcy-18(nj38) IV* were obtained from the Caenorhabditis Genetics Center. N2 Bristol was used as the wild-type reference strain.

### Genetic Screen and SNP Mapping

The *wy349* allele was isolated from an F2 semiclonal visual screen of approximately 3000 haploid genomes in the strain *syd-2(ju37)X*; *wyIs111 II*. Fifty mM EMS was used to mutagenize the worms. SNIP-SNP mapping and sequencing were executed using standard protocols.

### Cloning and Constructs

Expression constructs were generated in the pSM vector, a derivative of pPD49.26 (A. Fire, S. McCarroll, and C. I. Bargmann, personal communication). The following plasmids and transgenic strains were generated using standard techniques: *wyIs111(Pgcy-8::sad-1::YFP (4 ng)*, *Pgcy-8::mcherry::rab-3 (0.4 ng)*, *Pttx-3::cfp (40 ng))*, *wyEx2019(Pgcy-8::gfp::syd-2(cDNA) (4 ng))*, *wyEx2169(Pgcy-8::gfp::syd-2(cDNA) (4 ng)*, *Pgcy-8::mCherry::rab-3 (0.5 ng))*, *wyEx3406 (Pgcy-8::sad-1s::yfp(4 ng))*, *wyEx1039* (*Pgcy-8::GFP::rab-3 (0.3 ng)*, *wyEx2930(Pgcy-8::sng-1::yfp (2 ng)),wyEx4440 (Pgcy-8::sng-1::YFP (2 ng),Pgcy-8::mcherry::rab-3 (0.4 ng))*, *wyEx4179 (Pgcy-8::tax-4::SL2::mcherry (40 ng))*, wyEx795 (Pttx-3::cfp (20 ng), Pgcy-3::mcherry (20 ng)). The GenBank accession numbers for the cDNAs used are as follows: *sad-1*, AF316542; *syd-2*, AF170122; *rab-3*, NM_001026802; *sng-1*, NM_076838, *tax-4* NC_003281. All new data have been deposited in GenBank. The co-injection markers *Punc-122::rfp* or *Pttx-3::cfp* were injected at 20 ng/uL or 40 ng/uL, respectively. Transgenic lines were generated as previously described [24].

### Fluorescence Confocal Imaging and Quantification

Images of fluorescently-tagged fusion proteins were captured in live *C. elegans* using a Plan-Apochromat 63×1.4 objective on a Zeiss LSM 710 confocal microscope. Worms were immobilized using 10 mM levamisole (Sigma-Aldrich).

Quantification of AFD axons was performed by imaging at least 20 L4 hermaphrodites under identical image and laser settings for each genotype. Only animals whose left and right cell bodies were in the same position within the X-Y plane were used in order to minimize potential rotational effects. Confocal stacks were taken, from which a maximum intensity projection was derived using the Zeiss Zen software. All images were then converted into TIF files for further analysis in ImageJ. The “straighten to line” function in the EMBL suite of ImageJ was then used to obtain straightened axons from these images. Puncta number and intensity were calculated using the “analyze particles” function in ImageJ. The following thresholds for intensity and puncta size, respectively, were used: 25 and 2 for *wyEx1039*; 20 and 2 for *wyEx2930*; 10 and 3 for *wyEx2169*; 15 and 2 for *wyEx3406*. The position of each punctum was calculated using the ImageJ “Analyze Particles Centroid” measurement; the distance from the center of each punctum to the center of the dorsal-most punctum in the same axon determined each punctum's position along the X-axis. AFD montages were generated by aligning straightened images using the center of the dorsal-most punctum.

### Cell autonomy quantification

At least 50 L4 animals of each genotype for each marker were quantified by eye under an inverted compound microscope using a Plan-Apochromat 63×1.4 objective. The extrachromosomal array containing *Pgcy-8::tax-4::SL2::mCherry* was crossed into *tax-4* lines containing the RAB-3, SYD-2, and SAD-1 markers. The SL2 site is an internal ribosomal entry site that ensured the quantification of only those animals expressing *tax-4*. Genotype was confirmed by sequencing.

## Supporting Information

Figure S1 **Development of presynapses in AFD. (A) Number of RAB-3 puncta over development.** As animals progress through larval stages, *tax-4* and *tax-2* mutants have more RAB-3 clusters than wild type starting at L3. (B) Number of SYD-2 puncta over development. As animals progress through larval stages, the number of SYD-2 clusters increases. *tax-4* and *tax-2* mutants have more SYD-2 clusters than wild type throughout development. (C) Number of SAD-1 puncta over development. As animals progress through larval stages, *tax-4* and *tax-2* mutants have fewer SAD-1 clusters than wild type throughout development. ***, p<0.001 for *tax-4* compared to wild-type; ###, p<0.001 for *tax-2* compared to wild-type.
(TIF)

Figure S2 **Co-localization of RAB-3 and SNG-1.** (A) Trace and line scans of the axon of a representative wild-type L4 animal. Intensity peaks of the RAB-3 and SNG-1 markers are highly correlated. (B) Trace and line scans of the axon of a representative *tax-4* L4 animal. Intensity peaks of the RAB-3 and SNG-1 markers are highly correlated. (C) Trace and line scans of the axon of a representative *tax-4* L1 animal. Intensity peaks of the RAB-3 and SNG-1 markers are highly correlated.
(TIF)

Figure S3 **Co-localization of RAB-3 and SYD-2.** (A) Trace and line scans of the axon of a representative wild-type L4 animal. Intensity peaks of the RAB-3 and SYD-2 markers are highly correlated though there are occasional RAB-3 puncta that lack corresponding SYD-2 puncta. (B) Trace and line scans of the axon of a representative *tax-4* L4 animal. Intensity peaks of the RAB-3 and SYD-2 markers are highly correlated though there are occasional RAB-3 puncta that lack corresponding SYD-2 puncta.
(TIF)

Figure S4 **Axon trajectory of AFD and AIY.** (A) AFD and AIY axons labeled with cytoplasmic fluropohores in wild-type animals fasciculate together. (B) As in wild-type animals, AFD and AIY axons labeled with cytoplasmic fluorophores in tax-4 animals fasciculate together. Neither axon displays noticeable morphological abnormalities. Scale bar, 10 µm
(TIF)

Figure S5 **Total puncta number in different markers. Total number of RAB-3, SYD-2, and SAD-1 puncta in wild-type,** ***tax-4*** **, and** ***tax-2*** **animals quantified by confocal microscopy and subsequent image analysis.** ***, p<0.001; p<0.05 compared to wild-type.
(TIF)
