# Supplementary figures and images for: Sensory Transduction Channel Subunits, tax-4 and tax-2, Modify Presynaptic Molecular Architecture in C. elegans
Source: PLoS One. 2011 Sep 7;6(9):e24562. doi: 10.1371/journal.pone.0024562 (PMC3168524; doi:10.1371/journal.pone.0024562)

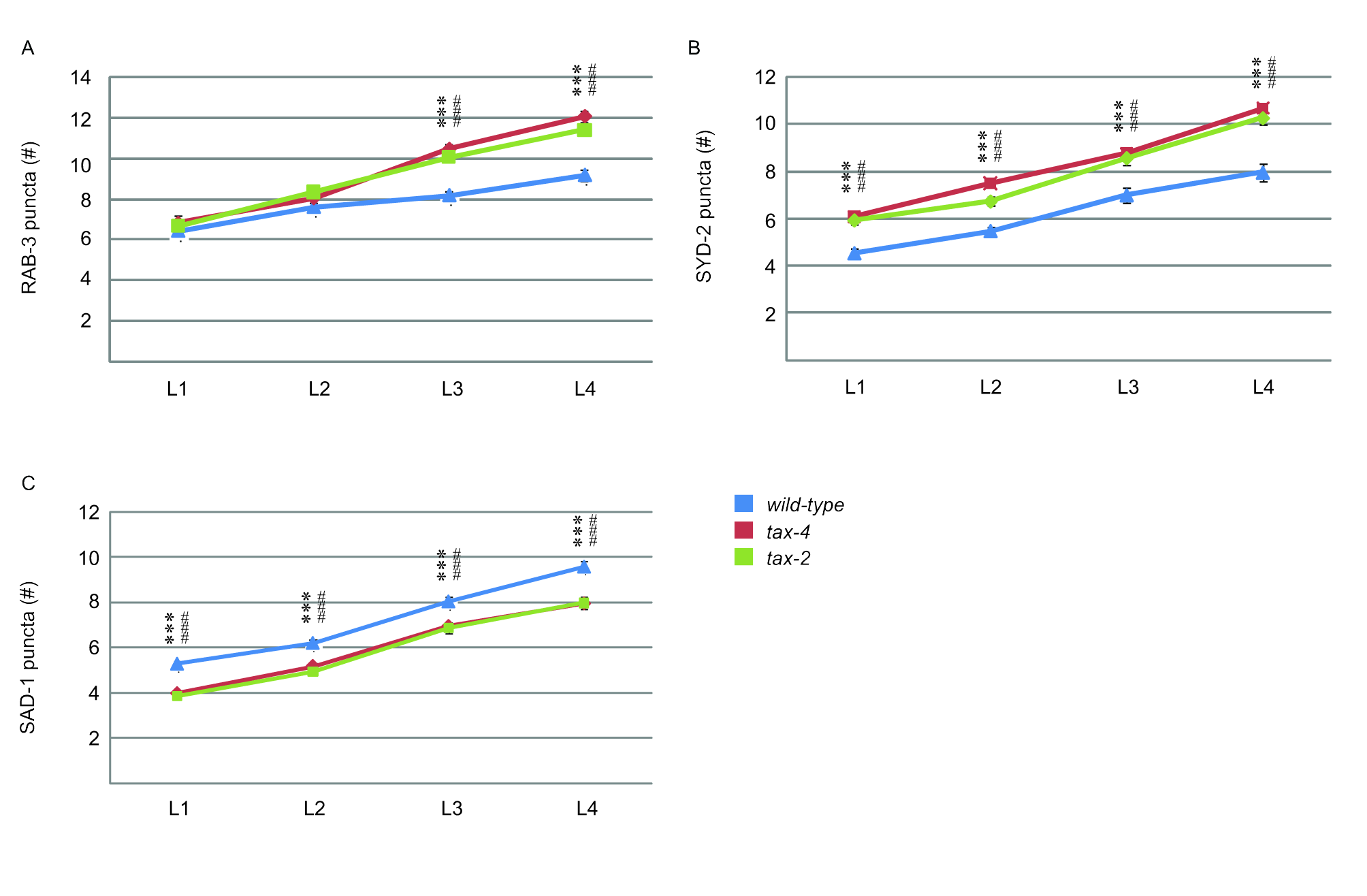

Supplement: Figure S1 — Development of presynapses in AFD. (A) Number of RAB-3 puncta over development. As animals progress through larval stages, tax-4 and tax-2 mutants have more RAB-3 clusters than wild type starting at L3. (B) Number of SYD-2 puncta over development. As animals progress through larval stages, the number of SYD-2 clusters increases. tax-4 and tax-2 mutants have more SYD-2 clusters than wild type throughout development. (C) Number of SAD-1 puncta over development. As animals progress through larval stages, tax-4 and tax-2 mutants have fewer SAD-1 clusters than wild type throughout development. ***, p<0.001 for tax-4 compared to wild-type; ###, p<0.001 for tax-2 compared to wild-type. (TIF) [file pone.0024562.s001.tif]

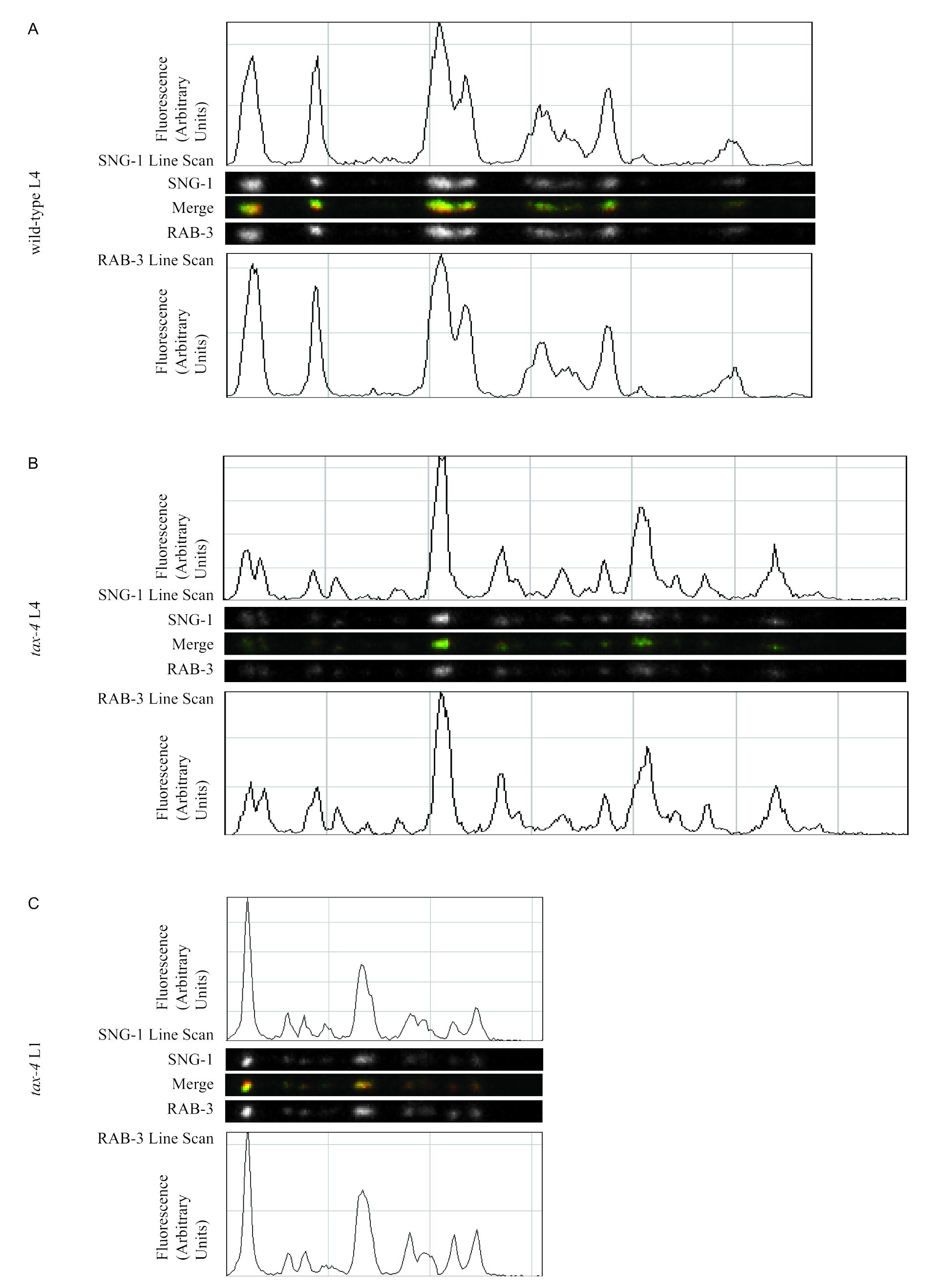

Supplement: Figure S2 — Co-localization of RAB-3 and SNG-1. (A) Trace and line scans of the axon of a representative wild-type L4 animal. Intensity peaks of the RAB-3 and SNG-1 markers are highly correlated. (B) Trace and line scans of the axon of a representative tax-4 L4 animal. Intensity peaks of the RAB-3 and SNG-1 markers are highly correlated. (C) Trace and line scans of the axon of a representative tax-4 L1 animal. Intensity peaks of the RAB-3 and SNG-1 markers are highly correlated. (TIF) [file pone.0024562.s002.tif]

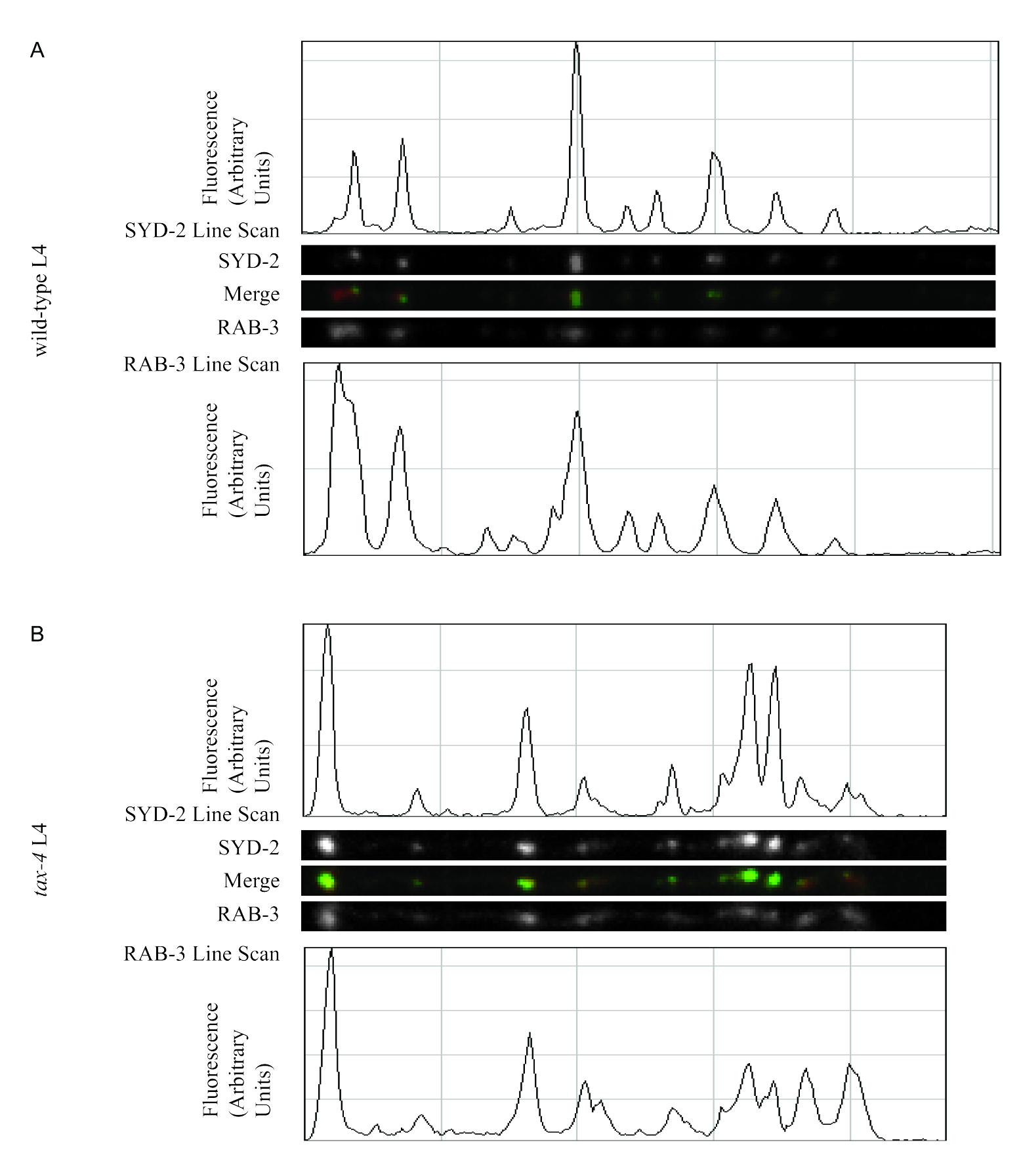

Supplement: Figure S3 — Co-localization of RAB-3 and SYD-2. (A) Trace and line scans of the axon of a representative wild-type L4 animal. Intensity peaks of the RAB-3 and SYD-2 markers are highly correlated though there are occasional RAB-3 puncta that lack corresponding SYD-2 puncta. (B) Trace and line scans of the axon of a representative tax-4 L4 animal. Intensity peaks of the RAB-3 and SYD-2 markers are highly correlated though there are occasional RAB-3 puncta that lack corresponding SYD-2 puncta. (TIF) [file pone.0024562.s003.tif]

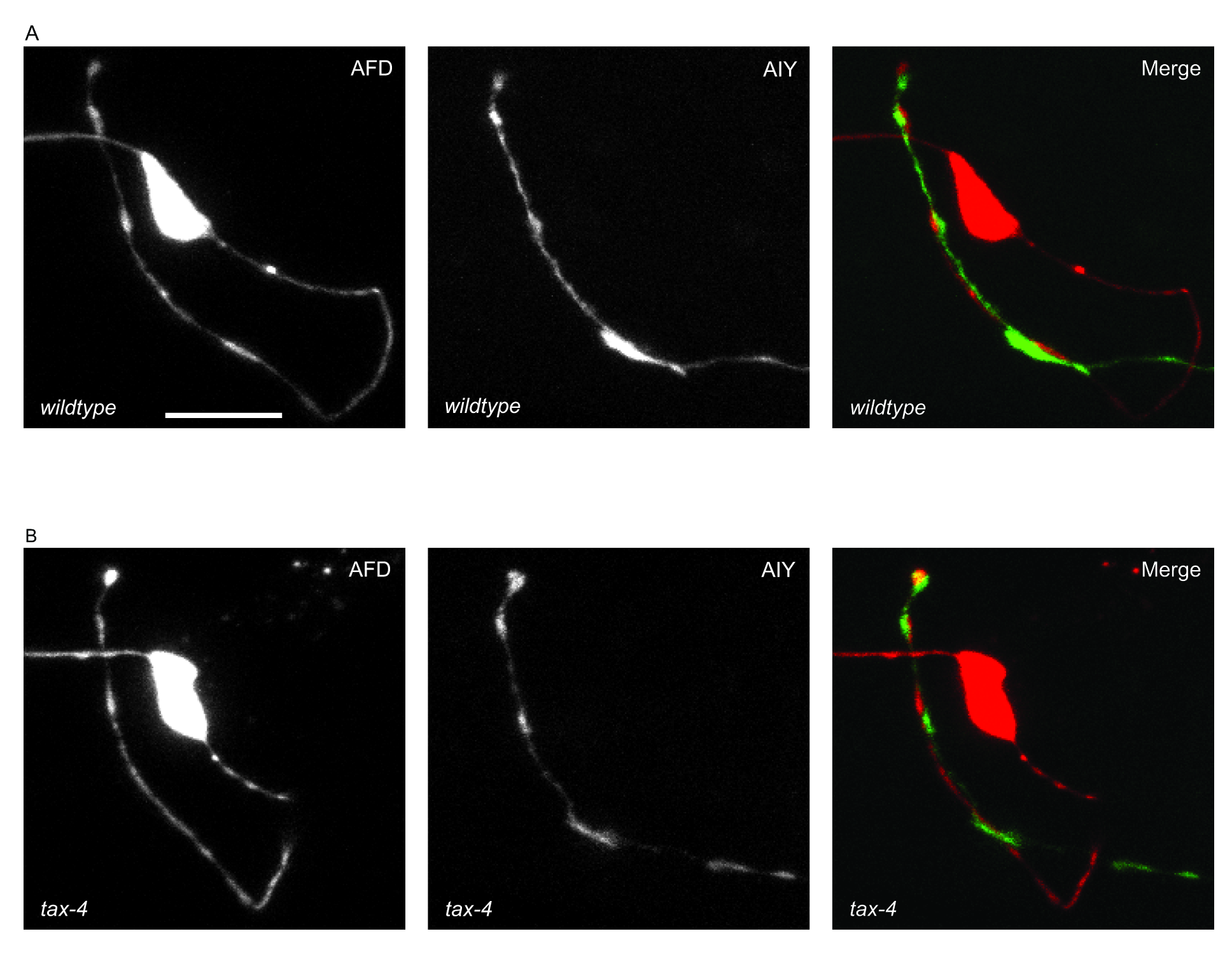

Supplement: Figure S4 — Axon trajectory of AFD and AIY. (A) AFD and AIY axons labeled with cytoplasmic fluropohores in wild-type animals fasciculate together. (B) As in wild-type animals, AFD and AIY axons labeled with cytoplasmic fluorophores in tax-4 animals fasciculate together. Neither axon displays noticeable morphological abnormalities. Scale bar, 10 µm (TIF) [file pone.0024562.s004.tif]

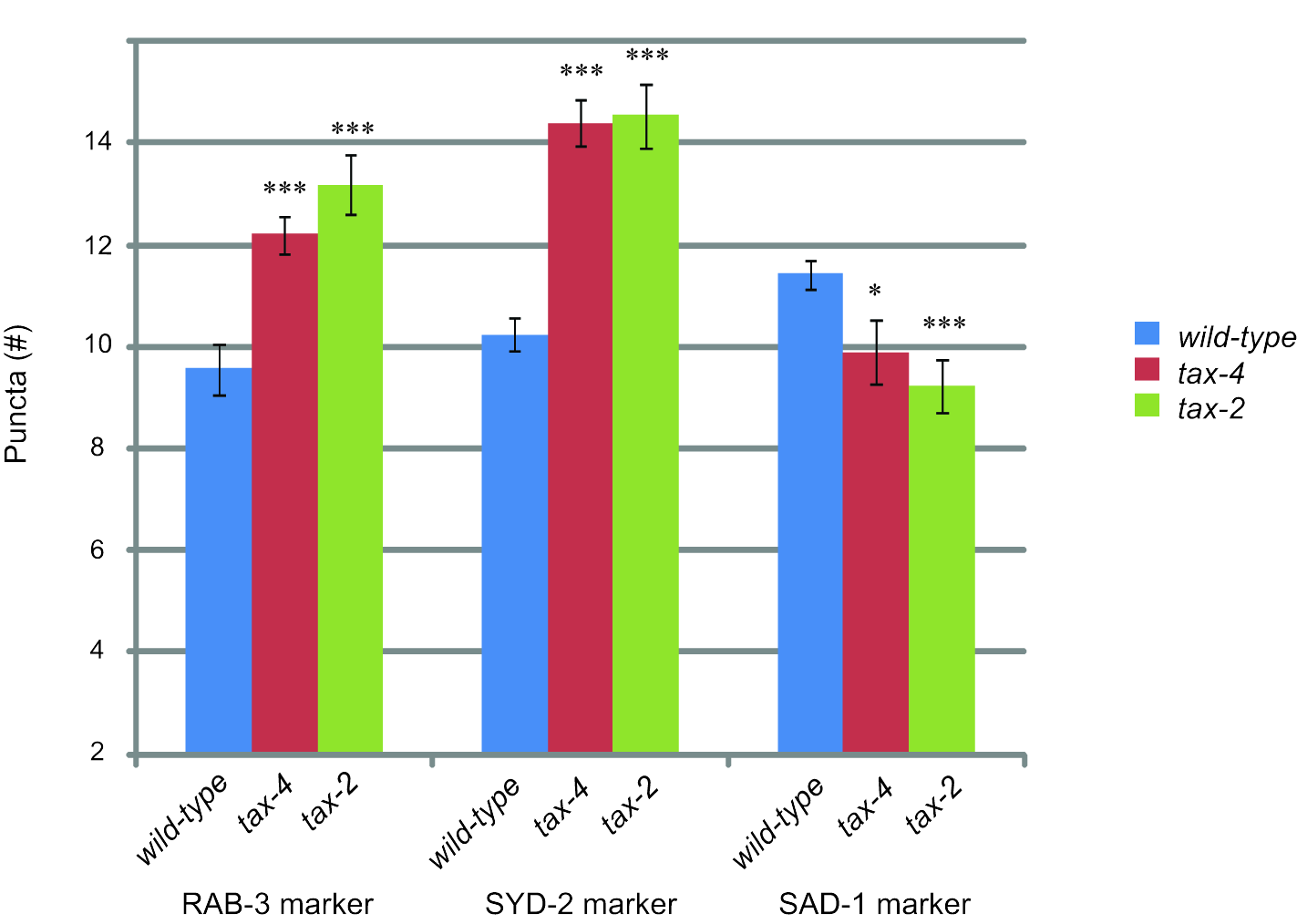

Supplement: Figure S5 — Total puncta number in different markers. Total number of RAB-3, SYD-2, and SAD-1 puncta in wild-type, tax-4 , and tax-2 animals quantified by confocal microscopy and subsequent image analysis. ***, p<0.001; p<0.05 compared to wild-type. (TIF) [file pone.0024562.s005.tif]
